# Cardioprotective Role of SIRT5 in Response to Acute Ischemia Through a Novel Liver-Cardiac Crosstalk Mechanism

**DOI:** 10.3389/fcell.2021.687559

**Published:** 2021-07-22

**Authors:** Boda Zhou, Min Xiao, Hao Hu, Xiaoxia Pei, Yajun Xue, Guobin Miao, Jifeng Wang, Wanqi Li, Yipeng Du, Ping Zhang, Taotao Wei

**Affiliations:** ^1^Department of Cardiology, Beijing Tsinghua Changgung Hospital, School of Clinical Medicine, Tsinghua University, Beijing, China; ^2^National Laboratory of Biomacromolecules, Institute of Biophysics, Chinese Academy of Sciences, Beijing, China; ^3^College of Life Sciences, University of Chinese Academy of Sciences, Beijing, China; ^4^Yuanpei College, Peking University, Beijing, China

**Keywords:** Sirt5, acute myocardial infarction, acylation, liver-cardiac crosstalk, FGF-21

## Abstract

Protein posttranslational modifications play important roles in cardiovascular diseases. The authors’ previous report showed that the abundance of succinylated and glutarylated proteins was significantly lower in the serum of patients with acute myocardial infarction (AMI) than in that of healthy volunteers, suggesting a potential relationship between protein acylation and AMI. Sirtuin 5 (SIRT5) facilitates the removal of malonyl, succinyl, and glutaryl modification; however, its effects on AMI remain unknown. In this study, the levels of SIRT5 in AMI mouse model was compared. Results showed elevated hepatic SIRT5 after myocardial infarction. Hepatocyte-specific SIRT5 overexpressing mice (liver SIRT5 OE) were generated to address the possible involvement of hepatic SIRT5 in AMI. The areas of myocardial infarction, myocardial fibrosis, and cardiac function in a model of experimental myocardial infarction were compared between liver SIRT5 OE mice and wild-type (WT) mice. The liver SIRT5 OE mice showed a significantly smaller area of myocardial infarction and myocardial fibrosis than the WT mice. The fibroblast growth factor 21 (FGF21) in the blood and myocardium of liver SIRT5 OE mice after AMI was markedly elevated compared with that in WT mice. The results of mass spectrometry showed increased levels of proteins regulating tricarboxylic acid cycle, oxidative phosphorylation, and fatty acid β-oxidation pathways in the liver mitochondria of liver SIRT5 OE mice. These findings showed that SIRT5 may exhibit a cardioprotective effect in response to acute ischemia through a liver-cardiac crosstalk mechanism, probably by increasing the secretion of FGF21 and the improvement of energy metabolism.

## Introduction

Lysine acylation is a large family of protein posttranslational modifications that use metabolic intermediates as group donors (Zhao et al., 2010; Lin et al., 2012). Acetylation occurring on histones facilitates the epigenetic regulation of gene expression, while acetylation on metabolic enzymes regulates the flux of metabolism (Zhao et al., 2010; Zhou et al., 2011). However, the physiological and pathophysiological functions of non-acetyl acylation have been under-investigated. Malonylation, succinylation, and glutarylation, which were found in histones (Xie et al., 2012) and metabolic enzymes (Du et al., 2015; Sadhukhan et al., 2016), use malonyl-CoA, succinyl-CoA, and glutaryl-CoA as substrates in their reaction, respectively (Hirschey and Zhao, 2015). Several groups have demonstrated the important roles of non-acetyl acylation in metabolism regulation in hepatic steatosis, diabetes, and heart failure (Rardin et al., 2013; Du et al., 2015, 2018; Sadhukhan et al., 2016), suggesting their potential importance in homeostasis and pathogenesis of metabolic disease. Recently we reported that the abundance of serum protein succinylation and glutarylation was significantly lower in the peripheral serum of patients with ST-elevation myocardial infarction and experimental acute myocardial infarction (AMI) rats than in healthy volunteers or sham surgery rats (Zhou et al., 2020). As the protein levels of cardiac SIRT5 were comparable before and after myocardial infarction, other mechanisms may be responsible for the removal of serum protein acylation during AMI. By profiling the expression of SIRT5 before and after myocardial infarction in mice, we found AMI resulted in elevated hepatic SIRT5 protein levels. Then we established a hepatocyte-specific SIRT5-overexpressing (liver SIRT5 OE) mouse model to investigate the pathophysiological functions of hepatic SIRT5 in AMI. Interestingly, the liver SIRT5 OE mice showed significantly smaller areas of myocardial infarction and myocardial fibrosis than the wild-type (WT) mice. Overexpression of SIRT5 increased the levels of blood and cardiac fibroblast growth factor 21 (FGF21) and elevated the expression of enzymes responsible for fatty acid β-oxidation in AMI mice, suggesting that SIRT5 may exhibit a cardioprotective role through a novel liver-cardiac crosstalk mechanism.

## Materials and Methods

### Antibodies and Reagents

Antibodies against SIRT5 (15122-1-AP), VDAC (10866-1-AP), Oxoglutarate Dehydrogenase L (OGDHL) (17110-1-AP), Hydroxyacyl-CoA Dehydrogenase Trifunctional Multienzyme Complex Subunit Alpha (HADHA) (1-758-1-AP), β-tubulin (10068-1-AP), α-tubulin (11224-1-AP), β-actin (66009-1-Ig), and HRP-conjugated α-tubulin (HRP-66031) were purchased from ProteinTech (Beijing, China). An antibody against FGF21 (ab171941) was from Abcam. An antibody against ATP synthase, H+ transporting, mitochondrial Fo complex, subunit d (ATP5H) (A4425) was from ABclonal. An anti-pan-succinyl-lysine (PTM-419) antibody was purchased from PTM Biolabs (Hangzhou, China). FGF21 ELISA kits were from R&D System (MF2100) and Abcam (ab212160). A list of chemicals used for mass spectroscopy could be found in the previously published paper (Du et al., 2015).

### Animals

Male C57BL/6J mice (12–16 weeks old, WT) were purchased from Vital River Co., Ltd. (Beijing, China). Hepatic SIRT5-overexpressing mice in C57BL/6 background (liver SIRT5 OE) was established by using the CRISPR/Cas9 gene editing tool described previously (Du et al., 2018). The males of this strain at the age of 12–28 weeks were used for the experiments. The mouse studies were approved by the Ethics Committee of Beijing Tsinghua Changgung Hospital (19032-0-01) and the National Health and Medical Research Council of China Guidelines on Animal Experimentation. All efforts were made to minimize animal suffering.

### Myocardial Infarction Experiments

Ligation of LCA in mice was performed as described previously (Lindsey et al., 2018). The mice were intubated and anesthetized with an animal anesthesia machine (Soft Lander; Sin-ei Industry Co., Ltd., Saitama, Japan). Left thoracotomy was performed to visualize the left auricle. LCA was ligated using a 7-0 suture (Ethicon, Inc., Somerville, NJ, United States). The muscle layers and skin were closed using a 3-0 suture. In sham-surgery mice, the same surgical procedures were performed without ligation of LCA. The body temperature of mice was maintained at 37°C with a heat lamp during the procedures. In total, 12 WT mice received myocardial infarction surgery (AMI-WT), 12 WT mice received sham surgery (Sham-WT), 12 liver SIRT5 OE mice received LCA ligation surgery at the same site as WT mice (AMI- SIRT5 OE), and 12 liver SIRT5 OE mice received sham surgery (Sham-SIRT5 OE). The peripheral blood was collected in all mice before and 24 h after surgery. Plasma or serum was isolated from whole blood and kept in −80°C until use. All mice were anesthetized and sacrificed 5 days after the surgery. Heart, liver, and other organs were sectioned.

### Histopathology

The heart was excised and sliced horizontally into 6–7 slices from base to apex. The morphological characteristics were analyzed via hematoxylin/eosin staining. Frozen heart sections (5 μm thickness) were fixed with acetone for 15 min, followed by Masson’s trichrome staining. The samples were incubated for 30 min in 1% 2,3,5-triphenyltetrazolium chloride (TTC) diluted in PBS (pH 7.4) at 37°C. The stained myocardial samples were visualized under a bright field microscope (Leica DM2500, Tokyo, Japan), and pictures of the entire slice were taken with identical exposure settings for all sections. The infarct/fibrotic area was compared with the total left ventricular (LV) area by using Image J software (NIH, Bethesda, MD, United States). The percentage of infarct area = infarct area/(infarct area + non-infarct area) × 100%, while the percentage of fibrotic area = fibrotic area/(fibrotic area + non-fibrotic area) × 100%.

### Echocardiography

Cardiac function was evaluated in the anesthesia. Ultrasound examinations were carried out using a high-resolution imaging system (Vevo 770, VisualSonics, Canada) with high-frequency ultrasound probe (RMV-707B, VisualSonics). M-mode images were obtained for measurements of LV wall thickness, LV end diastolic diameter (LVEDD), and LV end systolic diameter (LVESD). Ejection fraction (EF)% = LVEDV – LDESV/LVEDV × 100%, while fractional shortening (FS)% = (LVEDD – LVESD/LVEDD) × 100%.

### Western Blotting

Tissues were homogenized in 1 mL lysis buffer (50 mM Tris–Cl pH 7.4, 150 mM NaCl, 0.5 mM EDTA, 1 mM dithiothreitol, 1% Triton X-100, 0.5% sodium deoxycholate, and 0.1% sodium dodecyl sulfate). The homogenized tissues were centrifuged at 12,000 rpm, and the supernatant containing the tissue lysates were collected. The protein concentration was quantified with Pierce BCA Kit (Thermo Fisher Scientific). Western blot analysis was performed as described previously (Du et al., 2018).

### Measurements of FGF21

The FGF21 levels in mouse plasma were analyzed via ELISA with the use of Quantikine mouse FGF21 Immunoassay kit (R&D Systems) following the protocols provided by the manufacturer. The FGF21 levels in the culture medium of primary hepatocytes isolated from WT or SIRT5 OE mice were analyzed using an ELISA kit (Abcam) in accordance with the protocols provided by the manufacturer.

### Mass Spectrometry

Mitochondria were isolated from the liver of WT or liver SIRT5 OE mice, and proteins were extracted from the mitochondria following standard protocols (Du et al., 2018). The purified mitochondrial samples were disrupted by an ultrasonic processor on ice in lysis buffer (8 M urea/0.1 M Tris–HCl, pH 8.0) containing 1× Protease Inhibitor Cocktail (Roche). The protein solution was diluted 1:5 with 50 mM triethylammonium bicarbonate (TEAB) and digested with trypsin (1:50) at 37°C overnight. The digestion was desalted on OASIS HLB column, and peptides eluted with 60% acetonitrile were lyophilized via vacuum centrifugation. The dried peptides were dissolved with 100 mM TEAB buffer prior to labeling with tandem mass tags (TMTs). One hundred microgram of protein from each biological replicate of different experimental conditions was labeled with TMT 10plex (Thermo Fisher Scientific) in accordance with the manufacturer’s instructions.

All nano LC-MS/MS experiments were performed on Q Exactive (Thermo Fisher Scientific) equipped with an Easy n-LC 1000 HPLC system. The labeled peptides were loaded onto a 100 μm id × 2 cm fused silica trap column packed in-house with reversed phase silica (Reprosil-Pur C18 AQ, 5 μm, Dr. Maisch GmbH) and then separated on an a 75 μm id × 20 cm C18 column packed with reversed phase silica (Reprosil-Pur C18 AQ, 3 μm, Dr. Maisch GmbH). MS analysis was performed with Q Exactive mass spectrometer (Thermo Fisher Scientific). Under data-dependent acquisition mode, the MS data were acquired at a high resolution of 70,000 (m/z 200) across the mass range of 300–1600 m/z. The target value was 3.00E + 06, with a maximum injection time of 60 ms. The top 20 precursor ions were selected from each MS full scan with isolation width of 2 m/z for fragmentation in the HCD collision cell with normalized collision energy of 32%. Subsequently, MS/MS spectra were acquired at a resolution of 17,500 (m/z 200). The target value was 5.00E + 04, with a maximum injection time of 80 ms. The dynamic exclusion time was 40 s. The nano electrospray ion-source setting was as follows: spray voltage of 2.0 kV, no sheath gas flow, and heated capillary temperature of 320°C.

The raw data from Q Exactive were analyzed with Proteome Discovery version 2.2.0.388 using Sequest HT search engine for protein identification and Percolator for false discovery rate (FDR) analysis. FDR < 1% was set for protein identification. The peptide confidence was set as high for peptide filter. Protein quantification was also performed on Proteome Discovery 2.2.0.388 using the ratio of the intensity of reporter ions from the MS/MS spectra. Only the unique and razor peptides of proteins were selected for protein relative quantification. The co-isolation threshold was specified as 50%, and the average reporter S/N value should be above 10. Normalization mode was selected for the total peptide amount to correct experimental bias. The abundance values were normalized using Z-score to visualize the profiles of metabolic proteins in the hepatic mitochondria between the WT and liver SIRT5 OE mice. The normalized values were used to generate heat maps by using the pheatmap R-package in R (version 3.61).

### Statistical Analysis

Descriptive data were presented as the mean ± standard deviation (SD) for continuous variables. Data were analyzed using independent *t* test or one-way analysis of variance for continuous variables. Significance was assumed at a two-sided *p* value < 0.05. Statistical analysis was performed using GraphPad Prism (version 8.3.1).

## Results

### Generation of Liver SIRT5 OE Mice

**FIGURE 1:**
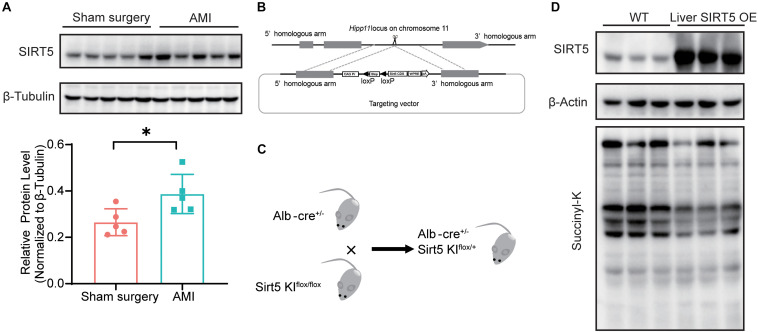
SIRT5 is up-regulated in the liver of experimental myocardial infarction mice. **(A)** Western blot analysis of hepatic SIRT5 in C57BL/6J mice 5 days after sham surgery or experimental myocardial infarction (AMI) (*n* = 5). Expression levels of SIRT5 were normalized to β-tubulin (*n* = 5). ^∗^*p* < 0.05 comparing with sham surgery. **(B)** Strategy of an alternative SIRT5 allele knock-in by CRISPR/Cas9 method. **(C)** Schematic diagram for breeding strategy to generate Liver SIRT5 OE mice. **(D)** Western blot analysis of SIRT5 and protein succinylation in the liver of WT mice and Liver SIRT5 OE mice (*n* = 3).

We recently reported decreased serum protein succinylation and glutarylation in patients and rats with AMI, but the levels of cardiac SIRT5 were comparable before and after myocardial infarction. Thus, the protein levels of SIRT5 in WT mice 5 days after AMI or sham surgery were compared. The results showed significantly elevated hepatic SIRT5 levels after AMI compared with sham surgery (Figure 1A). A hepatic specific SIRT5-overexpressing mouse model (liver SIRT5 OE) was established to further investigate the possible pathophysiological effects of hepatic SIRT5 in AMI. An alternative SIRT5 coding sequence was inserted into the Hipp 11 locus on the chromosome 11 of WT C57BL/6 mice via CRISPR/Cas9 method (Figure 1B). Hepatic SIRT5-overexpressing mice (liver SIRT5 OE) were generated by crossing between the SIRT5 allele knock-in mice (SIRT5 KI^*flox/flox*^) and Alb-cre mice (Alb-cre^±^), as shown in Figure 1C. Overexpression of SIRT5 in the liver but not in the kidney, brain, or heart was confirmed by Western blot (Figure 1D and Supplementary Figure 1). A marked decrease in total protein succinylation was found in the liver tissues of liver SIRT5 OE mice compared with that of WT mice (Figure 1D). Altogether, SIRT5 was specifically overexpressed in the liver of mice, and this overexpression significantly reduced levels of succinylation on multiple hepatic proteins.

### Hepatic Overexpression of SIRT5 Reduced Myocardial Infarction and Cardiac Fibrosis

**FIGURE 2:**
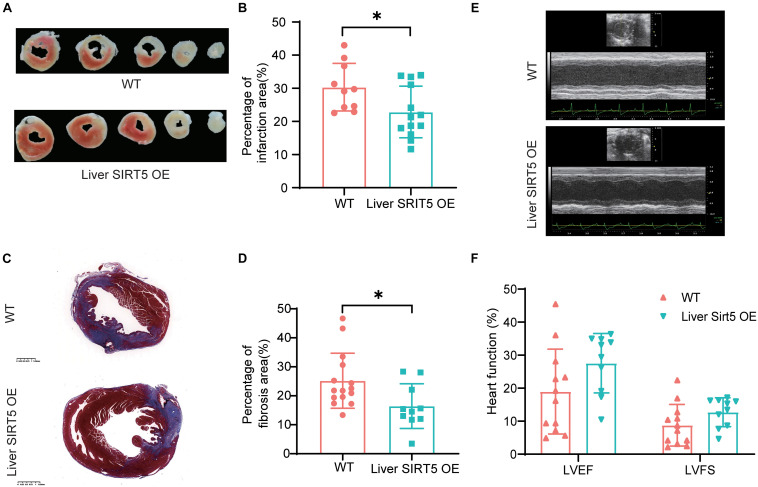
Comparation of infarction area between Liver SIRT5 OE and WT mice after AMI. **(A)** Representative images of triphenyltetrazole chloride (TTC) staining 5 days after AMI between Liver SIRT5 OE and WT mice (*n* = 6). **(B)** Comparation of infarction area 5 days after AMI between Liver SIRT5 OE and WT mice (*n* = 6). *p < 0.05. **(C)** Representative images of Masson staining 5 days after AMI between Liver SIRT5 OE and WT mice (*n* = 6). **(D)** Comparation of fibrosis area 5 days after AMI between Liver SIRT5 OE and WT mice (*n* = 6). *p < 0.05. **(E)** Representative images of echocardiography 4 days after AMI between Liver SIRT5 OE and WT mice (*n* = 6). **(F)** Comparation of heart function 4 days after AMI between Liver SIRT5 OE and WT mice (*n* = 6).

To understand the effect of hepatic overexpression of SIRT5 on myocardial infarction, we first compared the cardiac function by using echocardiography between liver SIRT5 OE and WT mice 4 days after experimental myocardial infarction. Although no significant difference was noticed before surgery (Supplementary Figure 2), we observed a trend toward higher (*p* = 0.081) LVEF in liver SIRT5 OE mice (27.55 ± 12.73%) compared with WT mice (18.97 ± 8.78%) after AMI, and a trend toward higher (*p* = 0.099) LVFS in liver SIRT5 OE mice (12.72 ± 4.36%) compared with WT mice (8.78 ± 6.29%) after AMI (Figures 2E,F). Second, the percentage of infarction area between the liver SIRT5 OE and WT mice 5 days after experimental myocardial infarction was compared using TTC staining. The results showed a significantly lower (*p* = 0.027) percentage of myocardial infarction in liver SIRT5 OE mice (22.84 ± 7.80%) than in WT mice (30.33 ± 7.20%, Figures 2A,B). Third, the percentage of fibrosis area between liver SIRT5 OE and WT mice 5 days after experimental myocardial infarction was compared using Masson staining. The results revealed a significantly lower (*p* = 0.019) percentage of fibrosis in liver SIRT5 OE mice (16.44% ± 7.71%) than in WT mice (25.17 ± 9.51%, Figures 2C,D).

### Hepatic Overexpression of SIRT5 Promoted Endocrine FGF21 From Liver

**FIGURE 3:**
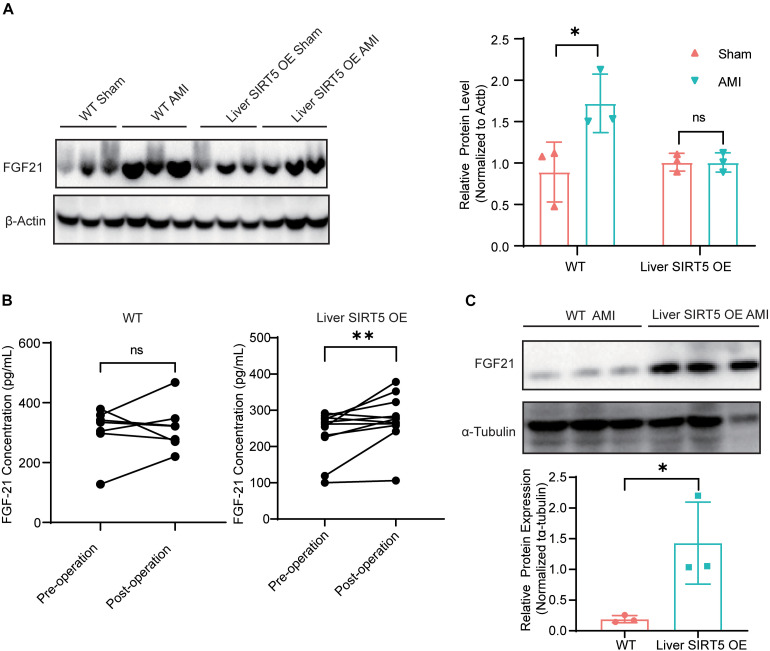
Expression and secretion of FGF21 in mice after AMI. **(A)** Western blot analysis of hepatic FGF21 in WT or Liver SIRT5 OE 5 days after sham surgery or AMI. Expression levels of FGF21 were normalized to β-tubulin (*n* = 3). ^∗^*p* < 0.05 comparing with sham surgery (*n* = 3). **(B)** Comparation of serum FGF21 level in mice before and after AMI (*n* = 5). **p < 0.01. **(C)** Western blot analysis of cardiac FGF21 in WT or Liver SIRT5 OE 5 days after sham surgery or AMI. Expression levels of FGF21 were normalized to β-tubulin (*n* = 3). ^∗^*p* < 0.05.

Fibroblast growth factor 21 played an important role in liver-cardiac crosstalk during myocardial ischemia by regulating energy expenditure. Therefore, we hypothesized that hepatic overexpression of SIRT5 may interfere with FGF21. The hepatic FGF21 protein level between sham surgery and AMI WT mice 5 days after surgery was detected, and the results showed a significantly elevated hepatic FGF21 protein level in AMI WT mice (Figure 3A). Surprisingly, a comparable hepatic FGF21 protein level was found in liver SIRT5 OE mice after sham surgery or AMI surgery, different from the results of WT mice (Figure 3A). Then, the blood FGF21 protein level between WT and liver SIRT5 OE mice before and after AMI surgery was compared. A significantly elevated blood FGF21 protein level was observed in liver SIRT5 OE mice after AMI surgery, whereas comparable blood FGF21 protein level was found in WT mice (Figure 3B). Interestingly, the cardiac but not muscular FGF21 protein level was significantly elevated in liver SIRT5 OE mice 5 days after AMI surgery compared with that in WT mice 5 days after AMI surgery (Figure 3C and Supplementary Figure 3A). Moreover, comparison of FGF21 secretion in the culture medium of primary hepatocytes isolated from liver SIRT5 OE or WT mice revealed significantly higher FGF21 level in hepatocytes from liver SIRT5 OE mice (Supplementary Figure 3B). These results suggested that the hepatic overexpression of SIRT5 promoted the secretion of FGF21 from the liver. There is also a possibility that hepatic overexpression of SIRT5 increased the expression or secretion of FGF21 from heart by potential mechanisms.

### Hepatic Overexpression of SIRT5 Improved Cell Metabolism

**FIGURE 4:**
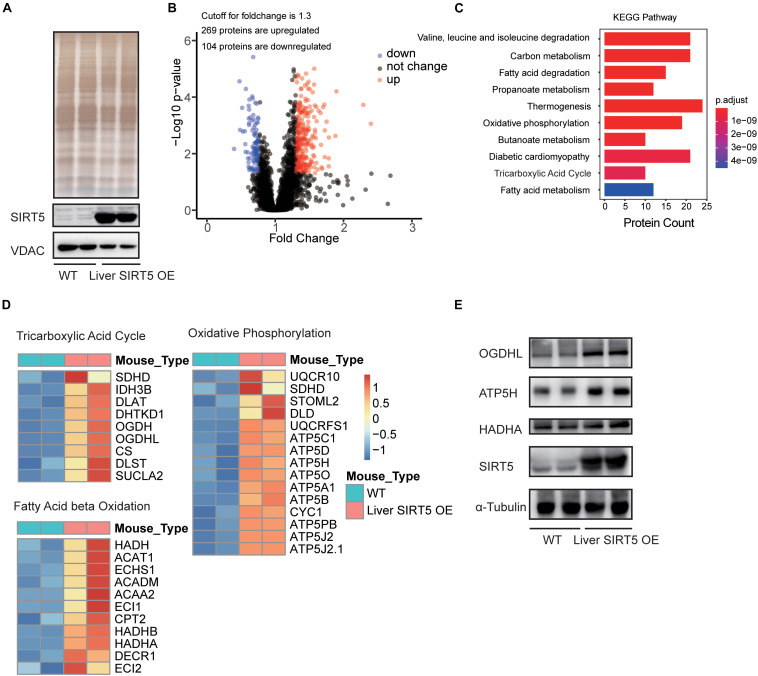
LC-MS analysis of hepatic mitochondria between Liver SIRT5 OE and WT mice. **(A)** Silver staining and Western blot of hepatic mitochondrial voltage-dependent anion channel (VDAC) and SIRT5 between Liver SIRT5 OE and WT mice in fasting state (*n* = 8). **(B)** Change distribution of peptides between Liver SIRT5 OE and WT mice. Red and blue dots indicate SIRT5 up-regulated and down-regulated peptides with the threshold of ratio (Liver SIRT5 OE/WT) ≥1.3 and *p*-value < 0.05 (*n* = 8). **(C)** Pathway enrichment of regulated proteins. **(D)** Heat map of regulated proteins involved in the tricarboxylic acid cycle, the fatty acid β-oxidation, and the oxidative phosphorylation. Blue or red colors represent downregulated or upregulated proteins, respectively (*n* = 8). **(E)** Western blot analysis of OGDHL, HADHA, and ATP5H and in Liver SIRT5 OE and WT mice (*n* = 2).

LC-MS/MS analysis of the hepatic mitochondria was performed to investigate the potential pathways regulated by the hepatic overexpression of SIRT5 (*n* = 8, Supplementary Figure 4). Western blot revealed significantly upregulated SIRT5 in the hepatic mitochondria isolated from liver SIRT5 OE mice (Figure 4A). Bioinformatic analysis reported 269 upregulated peptides and 104 downregulated peptides with at least 1.3-fold change in the hepatic mitochondria of liver SIRT5 OE mice (Figure 4B). Pathway enrichment analysis showed enrichment in the pathways regulating fatty acid metabolism, tricarboxylic acid cycle, and oxidative phosphorylation (Figure 4C). The heat map of the total hepatic mitochondrial proteins confirmed the significant upregulation of multiple proteins in fatty acid β-oxidation, tricarboxylic acid cycle, and oxidative phosphorylation pathways in liver SIRT5 OE mice compared with that in WT mice (Figure 4D), indicating the enhancement of mitochondrial metabolism. These results were verified by western blot and showed upregulation of OGDHL and ATP5H in the hepatic mitochondria isolated from liver SIRT5 OE mice (Figure 4E).

## Discussion

Cardiovascular diseases, especially myocardial infarction, have been the leading cause of death worldwide, even after modern therapeutic methods became prevalent, with ischemic injury secondary to energy shortage as the potential pathological basis (Pedersen et al., 2014). Activated upon caloric restriction, sirtuins control critical cellular processes in the nucleus, cytoplasm, and mitochondria to maintain metabolic homeostasis and reduce cellular damage to protect against ischemic injury (Winnik et al., 2015). SIRT5 has been reported to exhibit a protective role in the pathological process of AMI, as global knockout of SIRT5 increased the infarct size in a model of cardiac ischemia-reperfusion injury (Boylston et al., 2015) and developed heart failure (Sadhukhan et al., 2016). Surprisingly, the global knockout of SIRT5 (Hershberger et al., 2017) but not the cardiac-specific knockout of SIRT5 (Hershberger et al., 2018) resulted in increased mortality in response to pressure overload, thus suggesting that the cardioprotective role of SIRT5 is not dependent on the heart. The SIRT5 in other tissues and organs may also be responsible for the cardioprotection. In the present study, SIRT5 expression was profiled in an AMI mouse model, and significantly elevated SIRT5 levels were found in the liver of AMI mice, indicating a possible role of hepatic SIRT5 in AMI. To confirm the relationship between SIRT5 and AMI, a hepatic-specific SIRT5-overexpressing mouse model was established by using the CRISPR/Cas9-based transgenic strategy. The results showed a significantly smaller area of myocardial infarction and myocardial fibrosis in liver SIRT5 OE mice than in WT mice, suggesting a novel cardioprotective mechanism of SIRT5 through a liver-cardiac crosstalk mechanism.

Fibroblast growth factor 21 has been reported to play key roles via liver-cardiac crosstalk in response to AMI. Of note, Liu et al. (2013) demonstrated that FGF21 was upregulated and released from the liver and adipose tissues in myocardial infarction. In the present study, a significant elevation in the hepatic FGF21 levels of WT mice in response to AMI operation was also observed. FGF21 protected the myocardium via attenuation of apoptosis, prevention of oxidative stress, and inhibition of inflammation (Tanajak et al., 2015). The signaling pathway involved in the regulation of FGF21 expression was well-documented. In the liver, the SIRT1-mediated activation of FGF21 prevented liver steatosis caused by fasting (Jegere et al., 2014), indicating the possible relationship between sirtuins and FGF21. By using hepatocyte-specific SIRT5 overexpressed mice, the effects of SIRT5 in hepatic FGF21 were explored in AMI mouse models. Different from the case of WT mice, in which the hepatic FGF21 was elevated as a result of AMI operation, no apparent upregulation of FGF21 was observed in the liver SIRT5 OE mice after AMI operation. However, the blood and cardiac FGF21 levels in liver SIRT5 OE mice in response to AMI operation were significantly higher than those in WT mice. These data suggested that hepatic SIRT5 promoted the secretion of FGF21 from the liver into the circulation system, where it showed cardioprotective effects in AMI models.

Fibroblast growth factor 21 improved the energy supply to the heart by modulating the β-oxidation of fatty acid (Tanajak et al., 2015). Fatty acid oxidation is the major (50–70%) source of energy in the heart (Opie and Stubbs, 1976). During AMI, carbohydrate metabolism is temporarily disturbed. Instead, circulating free fatty acids are generally increased (Opie and Stubbs, 1976). LC-MS/MS analysis revealed an elevated expression of proteins regulating tricarboxylic acid cycle, oxidative phosphorylation, and fatty acid β-oxidation, the three essential pathways in the process of fatty acid oxidation, in liver SIRT5 OE mice. Western blot indicated that three proteins, OGDHL involved in the TCA cycle, ATP5H involved in the synthesis of ATP, and HADHA involved in the oxidation of fatty acids, were up-regulated in SIRT5 OE mice. This finding suggested that hepatic SIRT5 may accelerate fatty acid metabolism, which may promote energy supply to the heart during AMI. All these results were consistent with the findings observed in ob/ob mice (Du et al., 2018).

In conclusion, the results provided direct experimental evidence supporting a possible cardio-protective role of SIRT5 in response to acute ischemia. The increase in FGF21 secretion and the improvement of fatty acid metabolism may be crucial for the novel liver-cardiac crosstalk mechanism, which may shed light on the prevention and treatment of AMI and related heart diseases.

## Data Availability Statement

The original contributions presented in the study are included in the article/Supplementary Material, further inquiries can be directed to the corresponding authors.

## Ethics Statement

The animal study was reviewed and approved by the Ethics Committee of Beijing Tsinghua Changgung Hospital.

## Author Contributions

BZ, PZ, and TW initiated the study and developed the concept of the manuscript. BZ, MX, HH, YD, PZ, and TW designed the experiments. BZ, MX, HH, XP, YX, GM, JW, and WL performed the experiments. BZ, MX, PZ, and TW analyzed and interpreted the data and wrote the manuscript. All authors contributed to the article and approved the submitted version.

## Conflict of Interest

The authors declare that the research was conducted in the absence of any commercial or financial relationships that could be construed as a potential conflict of interest.

## Supplementary Material

Supplementary Figure 1 Western blot analysis of SIRT5 in the kidney, brain, and heart of WT mice and Liver SIRT5 OE mice (*n* = 3).

Supplementary Figure 2 Comparation of heart function before AMI. **(A)** Representative images of echocardiography before AMI between Liver SIRT5 OE and WT mice (*n* = 8). **(B)** Comparation of heart function before AMI between Liver SIRT5 OE and WT mice (*n* = 8).

Supplementary Figure 3 Detection of FGF21 in WT mice and Liver SIRT5 OE mice. **(A)** Western blot analysis of FGF21 in the muscle of WT mice and Liver SIRT5 OE mice (*n* = 3). **(B)** Primary hepatocytes were isolated from Liver SIRT5 OE or WT mice, and cultured *in vitro*. FGF21 protein was detected in the culture medium by ELISA and compared.

Supplementary Figure 4 Sample preparation for LC-MS analysis of hepatic mitochondria (*n* = 8): A total of eight WT c57 mice and eight Liver SIRT5 OE mice were subject to starvation for 12 h before experiment, then hepatic mitochondria were isolated from every four mice and processed as one sample in TMT-MS.
