# Supplementary figures and images for: Cardioprotective Role of SIRT5 in Response to Acute Ischemia Through a Novel Liver-Cardiac Crosstalk Mechanism
Source: Front Cell Dev Biol. 2021 Jul 22;9:687559. doi: 10.3389/fcell.2021.687559 (PMC8339556; doi:10.3389/fcell.2021.687559)

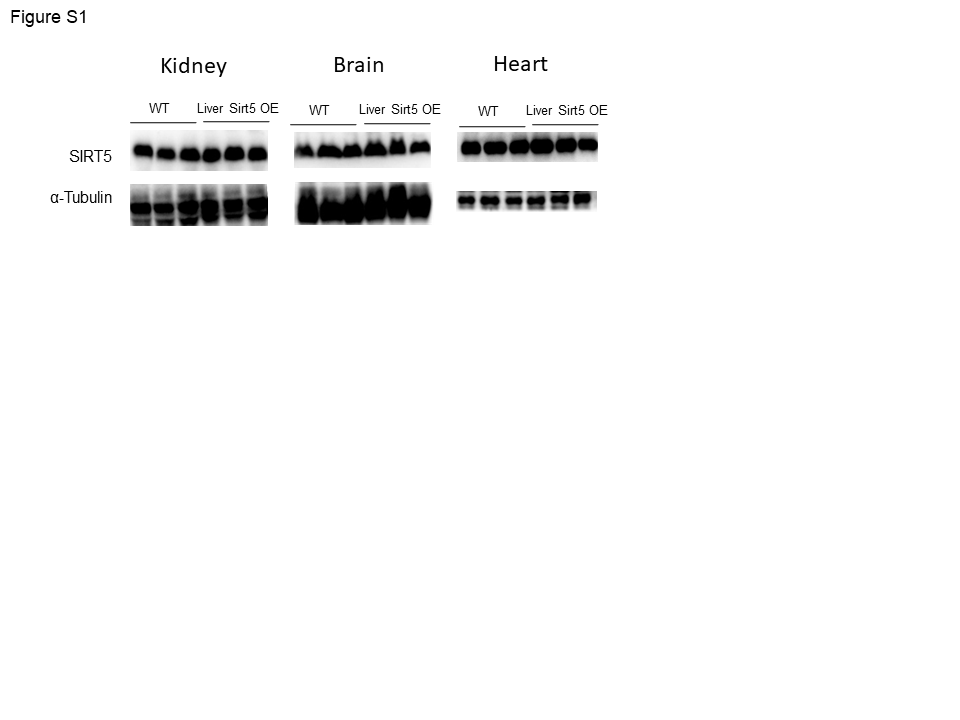

Supplement: Supplementary Figure 1 — Western blot analysis of SIRT5 in the kidney, brain, and heart of WT mice and Liver SIRT5 OE mice (n = 3). [file Image_1.TIF]

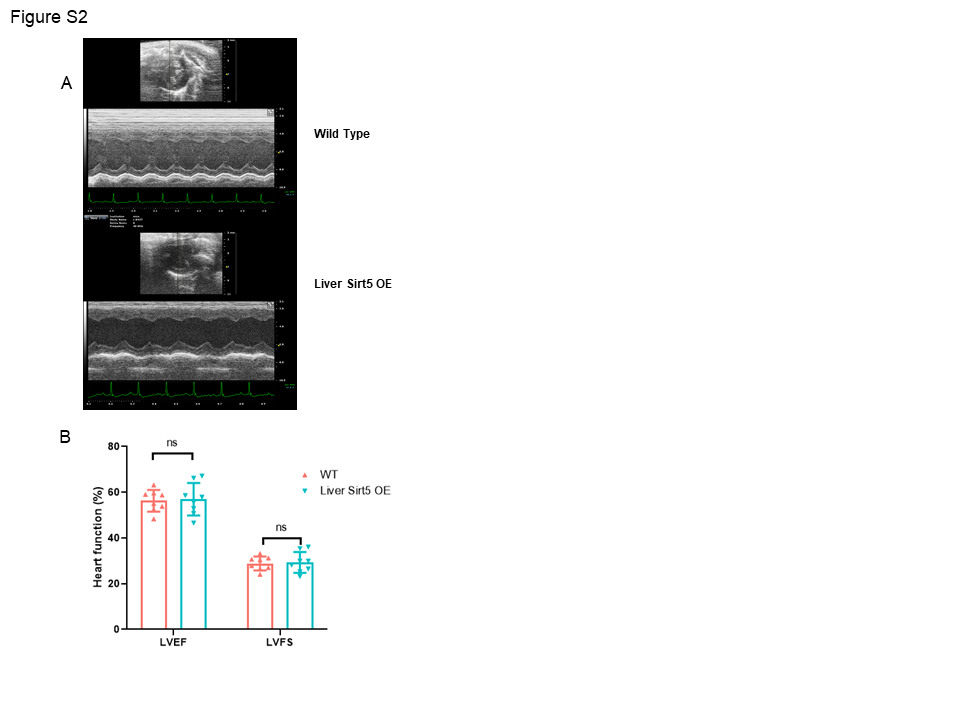

Supplement: Supplementary Figure 2 — Comparation of heart function before AMI. (A) Representative images of echocardiography before AMI between Liver SIRT5 OE and WT mice (n = 8). (B) Comparation of heart function before AMI between Liver SIRT5 OE and WT mice (n = 8). [file Image_2.TIF]

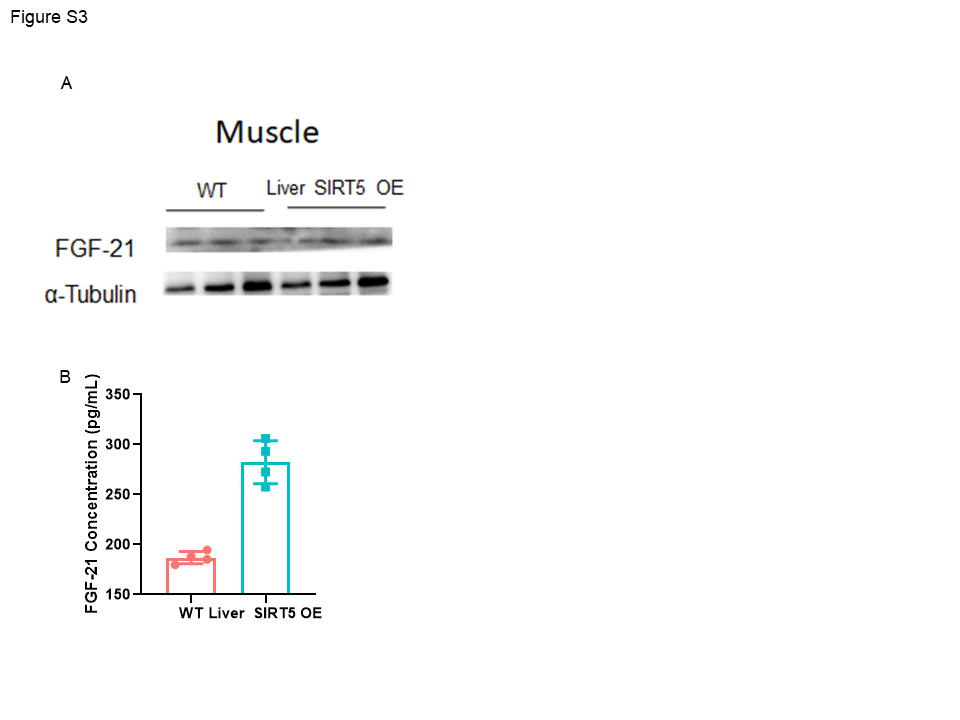

Supplement: Supplementary Figure 3 — Detection of FGF21 in WT mice and Liver SIRT5 OE mice. (A) Western blot analysis of FGF21 in the muscle of WT mice and Liver SIRT5 OE mice (n = 3). (B) Primary hepatocytes were isolated from Liver SIRT5 OE or WT mice, and cultured in vitro. FGF21 protein was detected in the culture medium by ELISA and compared. [file Image_3.TIF]

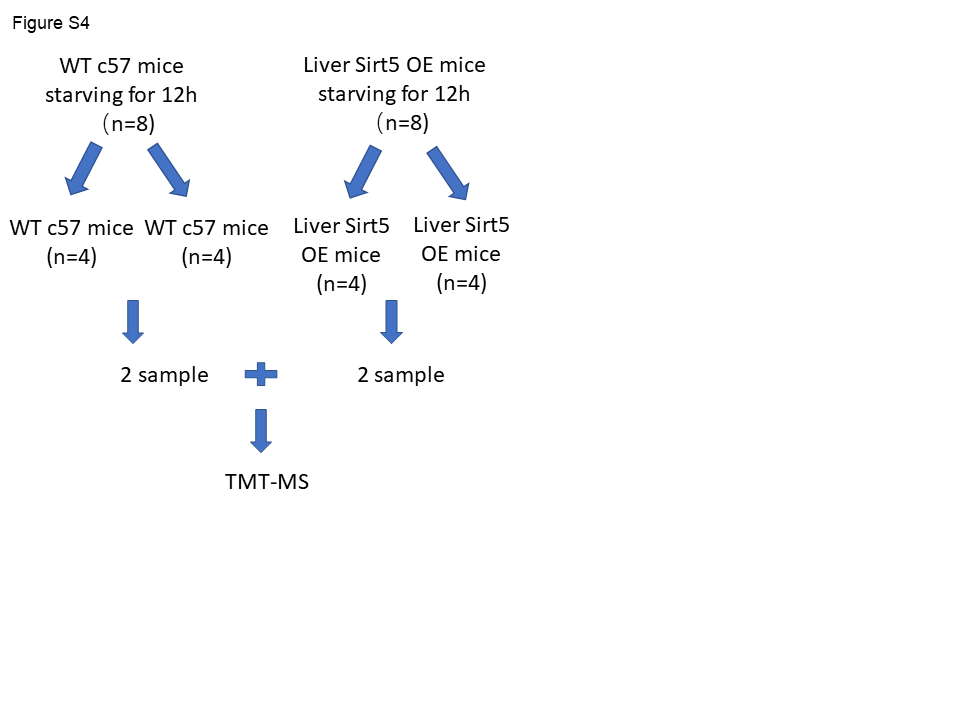

Supplement: Supplementary Figure 4 — Sample preparation for LC-MS analysis of hepatic mitochondria (n = 8): A total of eight WT c57 mice and eight Liver SIRT5 OE mice were subject to starvation for 12 h before experiment, then hepatic mitochondria were isolated from every four mice and processed as one sample in TMT-MS. [file Image_4.TIF]
